# The complete chloroplast genome of *Cicer reticulatum* and comparative analysis against relative *Cicer* species

**DOI:** 10.1038/s41598-023-44599-1

**Published:** 2023-10-19

**Authors:** Ezgi Mehmetoğlu, Yasin Kaymaz, Duygu Ateş, Abdullah Kahraman, Muhammed Bahattin Tanyolaç

**Affiliations:** 1 Faculty of Engineering, Department of Bioengineering, Ege University, 35100 Bornova, Izmir Turkey; 2 Faculty of Agriculture, Department of Field Crops, Harran University, S. Urfa, 64000 Şanlıurfa, Turkey

**Keywords:** Comparative genomics, Genomic analysis

## Abstract

The chloroplast (cp) genome is an adequate genomic resource to investigate evolutionary relationships among plant species and it carries marker genes available for species identification. The *Cicer reticulatum* is one of perennial species as the progenitor of cultivated chickpeas. Although a large part of the land plants has a quadruple chloroplast genome organization, the cp genome of *C. reticulatum* consists of one LSC (Large Single Copy Region), one SSC (Small Single Copy Region), and one IR (Inverted Repeat) region, which indicates that it has an untypical and unique structure. This type of chloroplast genome belongs to the IR-lacking clade. Chloroplast DNA (cpDNA) was extracted from fresh leaves using a high salt-based protocol and sequencing was performed using DNA Nanoball Sequencing technology. The comparative analysis employed between the species to examine genomic differences and gene homology. The study also included codon usage frequency analysis, hotspot divergence analysis, and phylogenetic analysis using various bioinformatics tools. The cp genome of *C. reticulatum* was found 125,794 bp in length, with an overall GC content of 33.9%. With a total of 79 protein-coding genes, 34 tRNA genes, and 4 rRNA genes. Comparative genomic analysis revealed 99.93% similarity between *C. reticulatum* and *C. arietinum*. Phylogenetic analysis further indicated that the closest evolutionary relative to *C. arietinum* was *C. reticulatum*, whereas the previously sequenced wild *Cicer* species displayed slight distinctions across their entire coding regions. Several genomic regions, such as *clpP* and *ycf1*, were found to exhibit high nucleotide diversity, suggesting their potential utility as markers for investigating the evolutionary relationships within the *Cicer* genus. The first complete cp genome sequence of *C. reticulatum* will provide novel insights for future genetic research on *Cicer* crops.

## Introduction

Chloroplasts are important vital organelles for plants. The main function of this organelle in plant cells is the implementation of energy metabolism known as photosynthesis^1,2^. It plays a role in the synthesis of metabolic units such as carbohydrates, lipids, and proteins, and the procurement of color pigments^3^. Chloroplast organelle has its own genomic DNA which makes it possible to regenerate independently in a plant cell^4^. This genome includes a certain number of genes with functions in the Photosystem I-II complex, cytochrome complex, NADH dehydrogenase, ATP synthase, RNA polymerases, RUBISCO, producing rRNAs and tRNAs, and ribosomal proteins^5–7^. Chloroplast genomes of land plants are typically formed as a quadrupole structure with one LSC, one SSC, and two IR regions^8,9^. The length of these regions varies according to the plant species, ranging between 60 to 200 Kb^10^. The IRLC (Inverted Repeat Lacking Clade) represents a diverse group of plants specifically from the Leguminosae family, that have undergone an evolutionary change in their chloroplast genomes^11^. This evolutionary change resulted in the loss of one of these IR regions (~ 25 kb). The IRLC comprises 56 genera, and approximately 4000 species that have experienced this evolutionary change, and *Cicer* genus taking part in this clade^12^.

The *Cicer* genus consists of 44 annual and perennial species^13,14^. These species form three different gene pools according to their crossability with the cultivated species, *Cicer arietinum*^15^. *Cicer reticulatum* belongs to the primary gene pool as the progenitor species, and it is reported to be native to the Southeastern part of Turkey^16^. The importance of this genus *Cicer* comes from its high protein, carbohydrate, and rich elemental content. Because of their favorable nutritional properties, members of this genus are widely used in human as well as animal nourishment. *C. reticulatum's* ability to crossbreed with the cultigen *C. arietinum* offers advantages to altering the cultivars in the *Cicer* genus through chloroplast (cp) genome engineering^17^. Likewise, cp genomes could be a practical tool to apply genetic engineering by modifying and improving key features on crops to better withstand challenging environmental conditions^18,19^. Furthermore, the cp genome exhibits a conserved structure, yet it has undergone numerous variations through the evolutionary processes. The diversity that occurs on the genome, enables the determination of phylogenetic relationships between species and facilitates species-oriented identifications. Thus, the chloroplast genome is important as a powerful tool for conducting precise and reliable phylogenetic analyses^20^.

Recent advancements in sequencing technologies have made organelle-based plant genetic and molecular biology research more accessible^21^. While the NCBI database contains complete chloroplast genomes of approximately four thousand plant species, only *Cicer arietinum*, *Cicer echinospermum,* and *Cicer bijugum* complete chloroplast genomes are available from the *Cicer* genus^22–24^. In this study, we aimed to: (i) reveal the whole chloroplast genome of *C. reticulatum* and its organization, (ii) conduct a comparative analysis of the *Cicer* genus and its relative species, (iii) examine the *Cicer* species’ evolutionary relationships based on cpDNA. Here, we present the first whole chloroplast genome sequence of the wild progenitor type, *C. reticulatum*, and their comparison with the available *Cicer* genus members and related species.

## Results

### Chloroplast genome organization and characteristics of *Cicer reticulatum*

**Figure 1:**
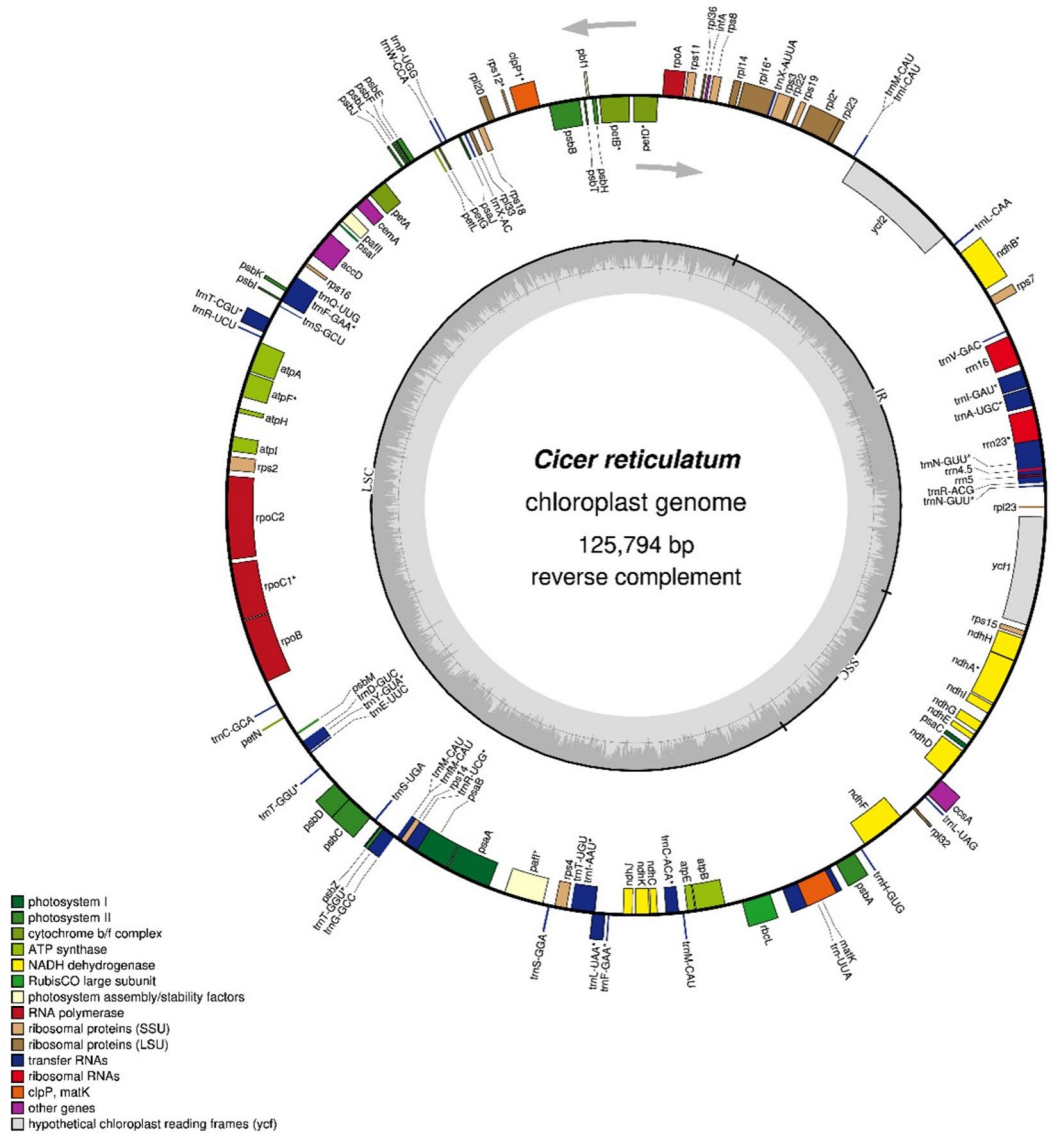
Circular chloroplast genome map of *Cicer reticulatum*. Genes inside the circle are transcribed in the clockwise direction, while genes outside the circle are transcribed in the counterclockwise direction. The dark grey area in the inner circle reflects the GC content of the cp genome, whereas the light grey area represents the AT content. The genes that belong to distinct functional groupings are shown by different color blocks.

The cp genome of *C. reticulatum* was found as 125,794 bp in size and the circular map of this genome is shown in Fig. 1. The genome showed a different structure from the other land plants and consisted of just one IR region (29,949 bp), one LSC region (82,531 bp), and one SSC region (13,314 bp) (Table 1). This genome organization belongs to the IRLC members which are known for the loss of one inverted repeat region. The GC content of the *C. reticulatum* cp genome was 33.9%. There were 117 genes found in the *C. reticulatum* cp genome (Table 2). Of these genes, 79 are protein-coding genes, 34 are tRNA genes, and 4 are rRNA genes. Among these, 23 genes (*ndhA, ndhB, rrn23, trnN-GUU, rps12, rpl2, rpl16, petD, petB, trnA-UGC, atpF, rpoC, trnI-GAU, trnF-GAA, trnT-CGU, trnY-GUA, trnT-GGU, trnR-UCG, trnI-AAU, trnL-UAA, trnC-ACA*) have one intron and 2 of them (*clpP1*, *ycf3*) have two introns. The largest intron, 2488 bp in length, was identified within the gene annotated as trnTERM-UUA, also known as trnK-UUU. Additionally, the smallest intron was 198 bp in length and was found within the rrn23 gene.

**Table 1:** Features of the chloroplast genome of *C. reticulatum, C. arietinum,* and *C. echinospermum*.

Species	*Cicer arietinum*	*Cicer reticulatum*	*Cicer echinospermum*
Size (bp)	125,319	125,794	126,713
LSC (bp)	82,528	82,531	83,129
SSC (bp)	13,038	13,314	13,288
IR (bp)	29,753	29,949	30,296
Genes	112	117	116
CDS	79	79	79
tRNA	29	34	33
rRNA	4	4	4
GC %	33.9%	33.9%	33.8%
AT %	66.1%	66.1%	66.2%

**Table 2:** Gene content and its functions of *C. reticulatum* chloroplast genome.

Category	Group of genes	Name of genes
	Large subunit of ribosomal proteins	rpl2^a^, rpl14, rpl16, rpl20, rpl22, rpl23, rpl32, rpl33, rpl36
	Small subuint of ribosomal proteins	rps2, rps3, rps4, rps7, rps8, rps11, rps12^a^, rps14, rps15, rpl16^a^, rps18, rps19
	DNA-dependent RNA polymerase	rpoA, rpoB, rpoC1^a^, rpoC2
Self-replication	Ribosomal RNA genes	rrn4.5, rrn5, rrn16, rrn23
		trnH-GUG, trnK-UUU, trnM-CAU, trnT-CGU^a^, trnT-GGU^a^, trnT-UGU, trnV-UAC, trnV-GAC
	Transfer RNA genes	trnF-GAA^a^, trnfM-CAU, trnL-UAA^a^, trnL-CAA, trnL-UAG, trnS-UGA, trnS-GCU, trnS-GGA
		trnG-UCC, trnG-GCC, trnE-UUC, trnY-GUA^a^, trnD-GUC, trnC-GCA, trnC-ACA^a^, trnR-UCU, trnR-ACG
		trnR-UCG^a^, trnQ-UUG, trnW-CCA, trnP-UGG, trnI-GAU^a^, trnI-AAU^a^, trnI-CAU, trnA-UGC^a^, trnN-GUU^a^
	Photosystem I	psaA, psaB, psaC, psaI, psaJ
	Photosystem II	psbA, psbB, psbC, psbD, psbE, psbF, psbH, psbI, psbJ, psbK, psbL, psbM, psbN (pbf1), psbT, psbZ
Genes for photosynthesis	RUBISCO	rbcL
	Subunits of ATPsynthase	atpA, atpB, atpE, atpF^a^, atpH, atpI
	Subunit of NADH- dehidrogenase	ndhA^a^, ndhB^a^, ndhC, ndhD, ndhE, ndhF, ndhG, ndhH, ndhI, ndhJ, ndhK
	Cytochrome b/f complex	petA, petB^a^, petD^a^, petG, petL, petN
	Protease	clpP^b^
	Maturase	matK
Other genes	Envelope membrane protein	cemA
	Translation initiation factor	infA
	C-type cytochrome synthesis gene	ccsA
	Subunit of Acetyl-CoA-carboxylase	accD
Genes of unknown function	Conserved hypothetical chloroplast	ycf1, ycf2, ycf3^b^ (pafI), ycf4 (pafII)

^a^one intron containing genes.

^b^two intron containing genes.

### Comparative genomic analysis

The differentiation level of the genes in the mVISTA analysis is associated with the white peaks in Supplemental Fig. 1. These differentiations can name nucleotide variations in specific genes or specific regions. As in other land plants, among these three genomes, variations that occurred in non-coding regions were greater than in the coding regions. Some genes such as *rps15, ycf1, ndhA, ndhH, ycf2,* and *accD* showed high nucleotide variations, which suggested potential marker regions. Moreover, MAUVE alignment was utilized to obtain gene homology between the species (using *C. arietinum* as a reference) and it showed that all the *Cicer* members had nearly the same gene order and structural organization, especially *C. reticulatum* and *C. arietinum* (Supplementary Fig. 2).

Among the species, four different LCB regions (Locally Colinear Blocks) were observed. Each histogram within these regions shows pairwise nucleotide sequence identity. MegaBLAST was performed to calculate the percentage similarity of the chloroplast genome sequences of these species with each other. It was determined that there was a 99.93% similarity between *C. arietinum* and *C. reticulatum*, a 99.79% similarity between *C. arietinum* and *C. echinospermum*, and a 99.80% similarity between *C. reticulatum* and *C. echinospermum*.

### Codon usage frequency and amino acid abundance

**Figure 2:**
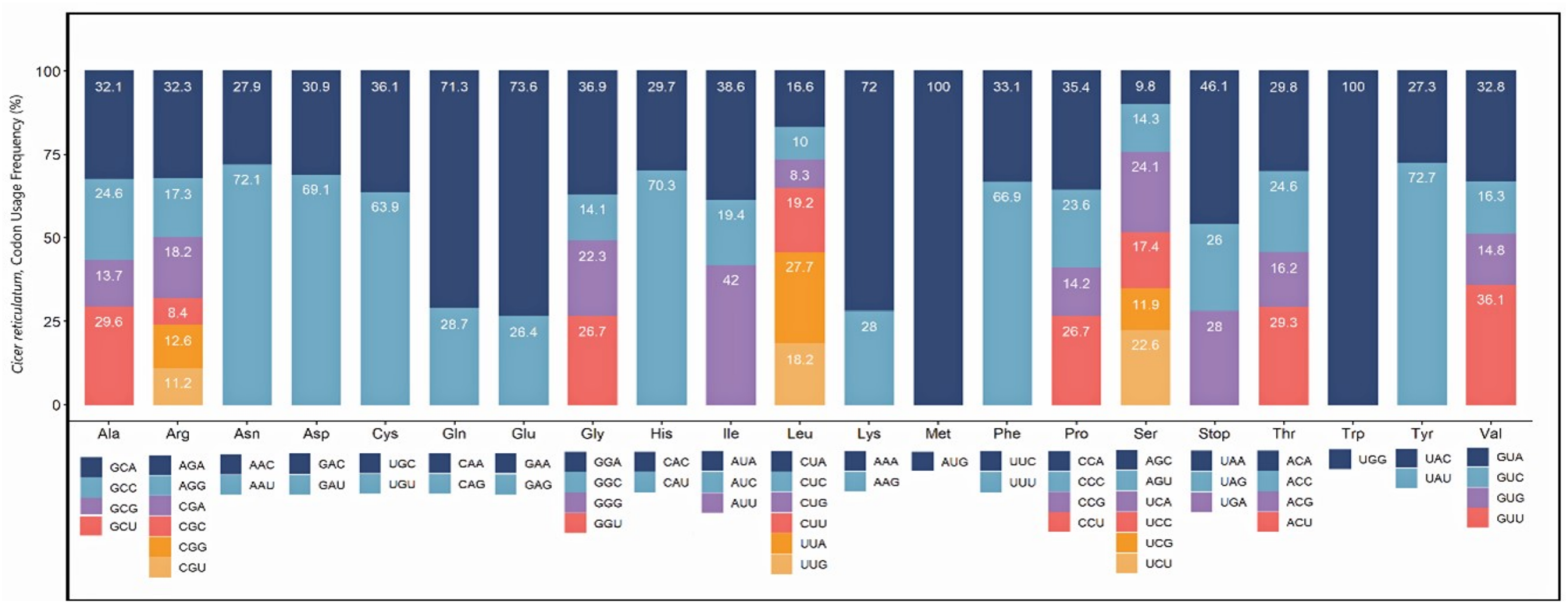
The codon usage dissemination of amino acids that are synthesized from the *Cicer reticulatum’s* chloroplast genome. The y-axis shows the proportion of codons that are used, while the x-axis shows the codons.

**Figure 3:**
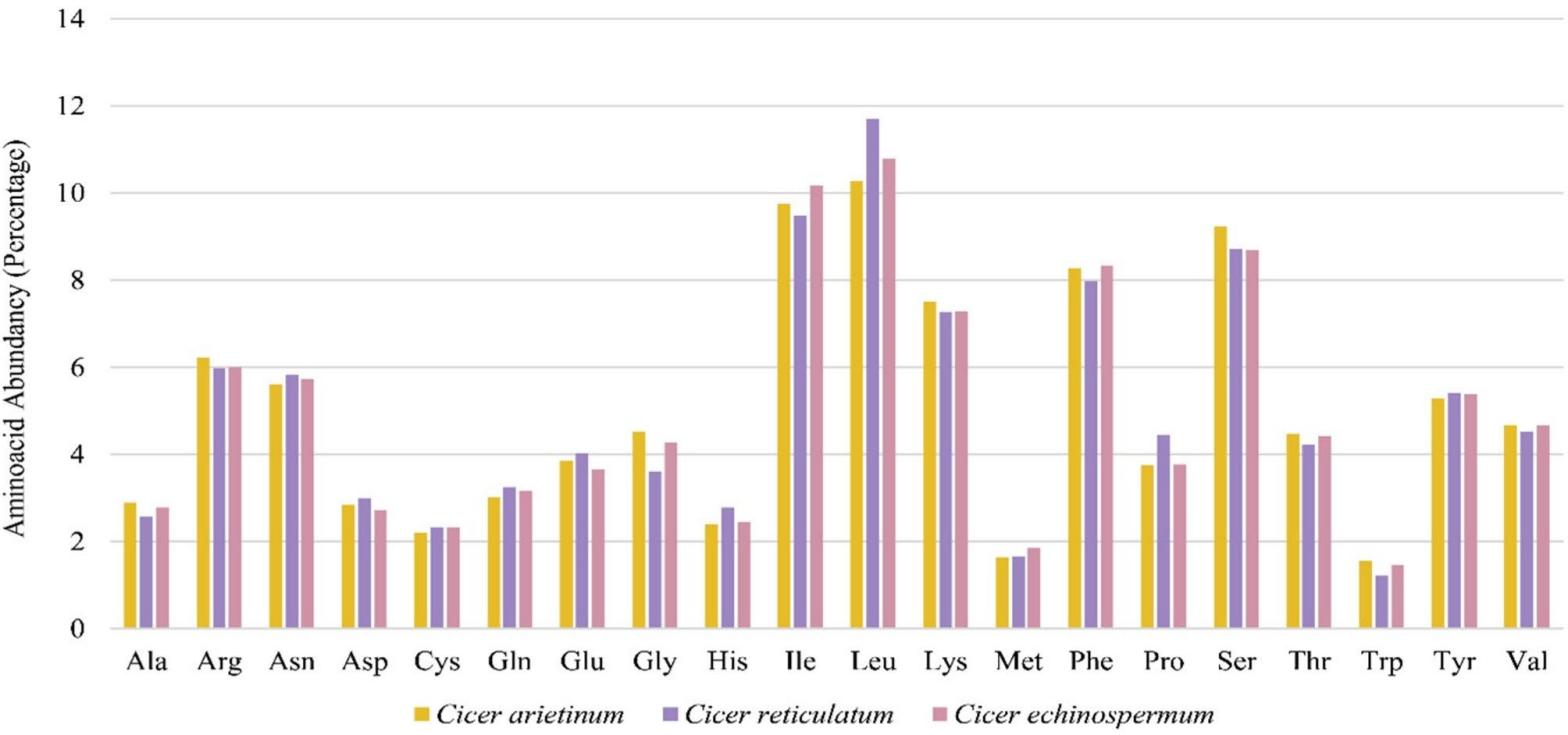
Three Cicer species chloroplast genome's amino acid distribution. The percentage abundances of each amino acid are shown on the x-axis.

A total of 41.931 codons were detected in the whole chloroplast genome sequence of *C. reticulatum* and their ratio by amino acids was given in Fig. 2. The most abundant codons belong to the phenylalanine amino acid (UUU) and lysine (AAA) amino acids with the percentage of 4.99% and 4.90%, respectively. The least abundant number of codons belong to the Alanine (GCG) amino acid with a percentage of 0.33%. Also, the RSCU (Relative synonymous codon usage) values of *C. reticulatum* codons were calculated. 31 codons showed strong bias (RSCU > 1) and they mostly carried A and U bases in their third position. 30 codons that showed weak bias (RSCU < 1) more often carried C and G in their third position. Besides, three codons which are AUG, UGG, and CUA had no bias (RSCU = 1) or codon usage preferences (Supplementary Table 1). In addition to these, the most abundant amino acids in the *C. reticulatum* cp genomes were found as leucine (11.6%), isoleucine (9.4%), and serine (8.7%). Tryptophan (1.22%) was found to be the least represented amino acid. A comparison of amino acid contents of all three *Cicer* species is given in Fig. 3.

### Repetitive sequence analysis

**Figure 4:**
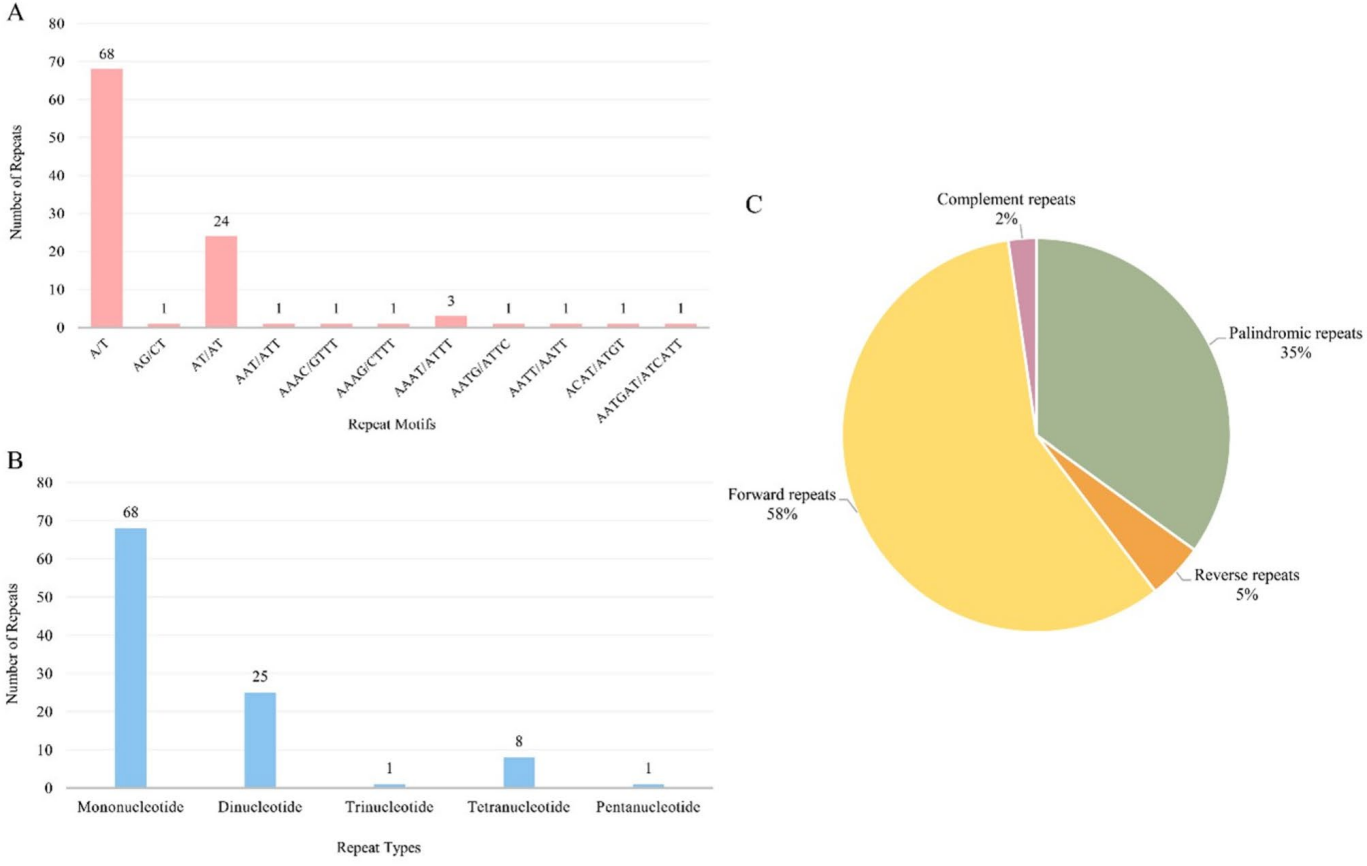
In the *Cicer reticulatum* chloroplast genome, the frequency of simple sequence repeats (SSRs) and repetitive sections. (**A**) The number of distinct types of SSRs. (**B**) The total number of SSR motifs. (**C**) Repetitive region distribution.

In the *C. reticulatum* genome, there were a total of 103 SSRs comprising 68 mononucleotides, 25 dinucleotides, 1 trinucleotide, 8 tetranucleotides, and 1 pentanucleotide. From the point of SSR motifs, the most common types in this genome were the A/T mononucleotide motif (68) and AT/AT dinucleotide motif (24). Contingent on REPuter-web tool results, there were 50 forward, 30 palindromic, 4 reverse, and 2 complement repeats found in this cp genome. These repeats were found in a wide range of base pairs; forward repeats between 35 and 285 bp, palindromic repeats between 30 and 133 bp, reverse repeats between 31 and 34 bp, and complement repeats between 30 and 31 bp (Fig. 4).

### Divergent hot spot analysis

**Figure 5:**
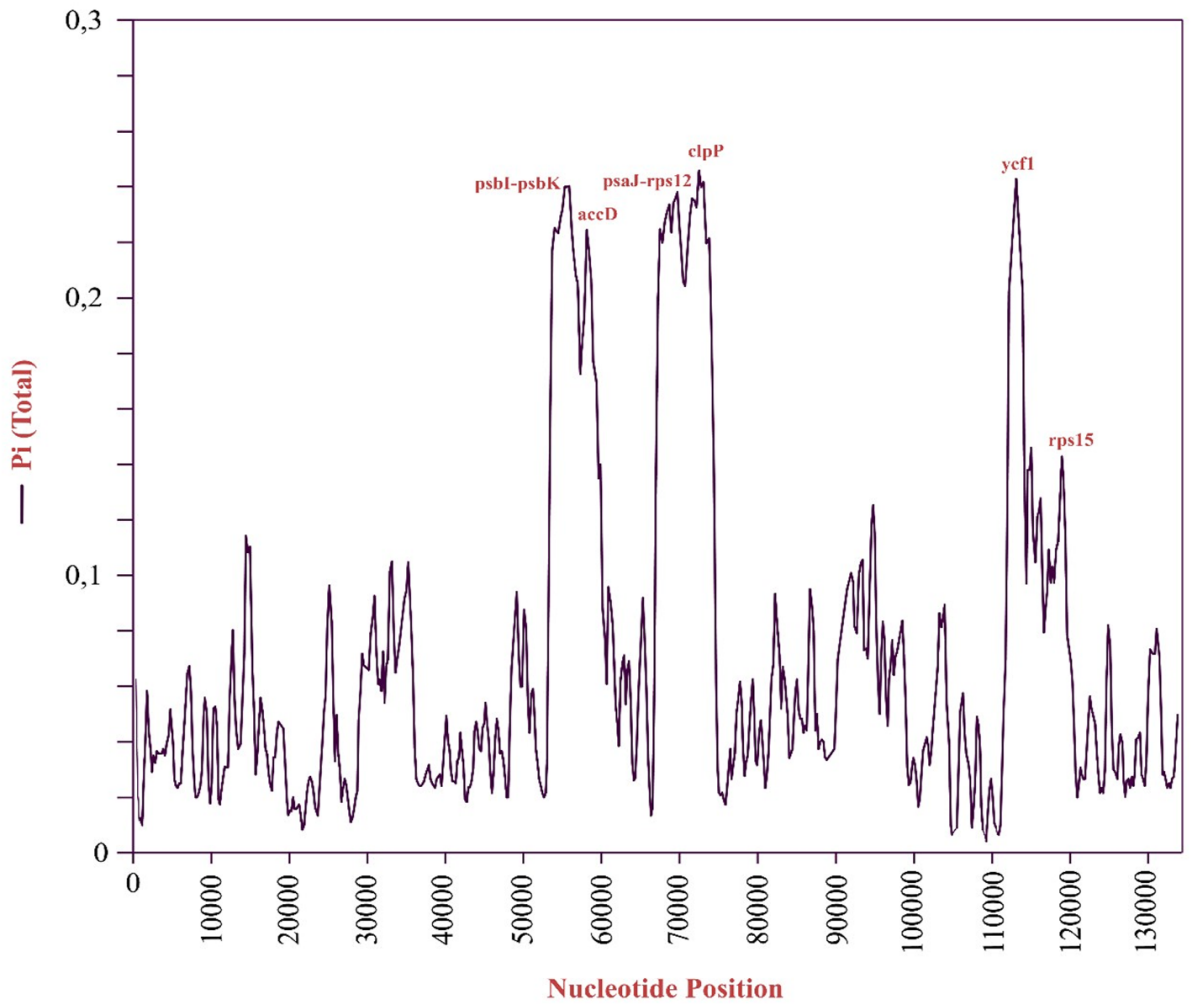
Sliding window analysis among the whole chloroplast genome of *Cicer reticulatum, Cicer echinospermum, Cicer arietinum,* and *Lens culinaris*. The X-axis shows the whole cp genome in 10 kb increments, while the y-axis shows the nucleotide diversity of genomes. Window length: 600 bp, step size: 200 bp.

Nucleotide diversity analysis shows certain nucleotide variability between the genomes and offers to determine molecular markers with the identification of highly divergent regions. To determine the nucleotide diversity in chloroplast genomes, sliding window analysis was performed with the DNAsp v6.11.01. Program among four different species which are *C. reticulatum, C. arietinum, C. echinospermum,* and *Lens culinaris*. As demonstrated in Fig. 5, the nucleotide variability (π) ranged from 0.24583 to 0.00417. The most divergent regions between these genomes were found as the genes *psbI-psbK, accD, psaJ-rps12*, *clpP, ycf1,* and *rps15* (Pi > 0.1133). The highest nucleotide variability is seen in the *clpP* and *ycf1* genes, with the 0.24583 and 0.24278 pi ratios, respectively.

### Phylogenetic analysis

**Figure 6:**
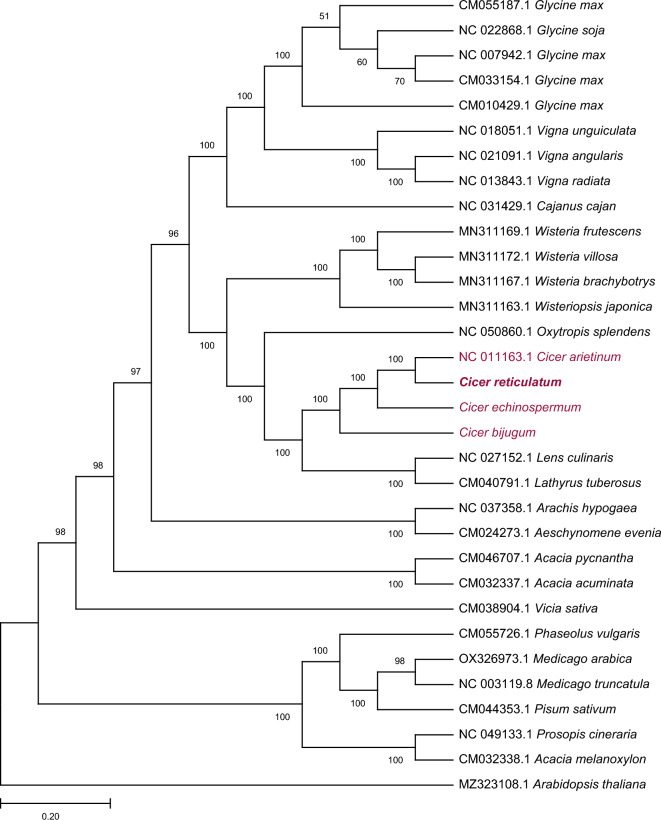
Phylogenetic tree reconstruction of 32 different cp genomes. The General Time Reversible (GTR) model and Gamma distribution were used in a Maximum Likelihood (ML) analysis using 1000 bootstrap repetitions.

In the phylogenetic analysis of 28 available chloroplast genomes from Leguminosae, the *Cicer* species formed a separate group closer to *Lens culinaris* and *Lathyrus tuberosus* (Fig. 6). *C. arietinum* and *C. reticulatum* showed a slightly close relationship while *C. echinospermum* and *C. bijugum* were relatively diverged away as expected. The tree contained a larger subgroup of species with multiple representatives, such as *Cicer*, *Wisteria*, *Vigna*, *Glycine,* etc. The divergence distance between the species of this group was evenly distributed, as supported by their branch lengths. On the other hand, a relatively smaller group of species also formed a separate clique with members such as *Phaseolus*, *Medicago*, *Pisum*, *Prosopis*, and *Acacia*. The distances between these two groups of species and a few individual branching directions suggest an early divergence and distant relationship among Leguminosae members that are examined here.

## Discussion

The chloroplast organelle maintains its genetic material as a circular genome known as cpDNA, which performs autonomously from the nuclear genome, shows self-replication capability, and remains conserved throughout the evolutionary process^25–28^. Due to genomic rearrangements and gene/intron losses, especially IR-loss, in the chloroplast genome structure of the Papilionoideae subfamily including the *Cicer* genus, species in this subfamily are classified as IRLC (Inverted Repeat Lacking Clade)^29–31^. Our analysis of the *C. reticulatum* chloroplast genome reveals a ~ 25 kb inverted repeat (IR) loss, which leads to a total genome length of 125,794 bp. Importantly, the chloroplast genome structure and content of both wild *Cicer* species, *C. reticulatum* and *C. echinospermum*, closely resemble that of *C. arietinum*, with the same IR loss observed in these wild species. These results align with previous reports regarding the genome structure^32,33^.

A chloroplast genome comparison of three different *Cicer* species was done with the mVISTA tool to detect major genomic rearrangements that occur at the base level, as shown in Supplementary Fig. 1. The chloroplast genomes of two wild *Cicer* species (*C. reticulatum* and *C. echinospermum*) were found to be quite similar to the cultured type *C. arietinum*'s chloroplast genome. The level of variations in certain regions peaked in the non-coding regions of the genomes^34,35^. Among these three genomes, exonic regions such as *rps15, ycf1, ndhA, ndhH, ndhF, atpB, atpE, rpoC,* and *accD* showed a high level of variations. The largest LCB region is about 104 kb in length, while the smallest LCB region is about 560 bp in length and is shown in Supplementary Fig. 2 as a red block and yellow block, respectively. There is one major inversion in the *C. echinospermum* cp genome, formed by reversing the ~ 21 kb long region shown as the blue block in Supplementary Fig. 2. This inversion is the most notable point among these three cp genomes. The fact that this inversion occurs naturally in the evolutionary process, is also supported by additional in silico alignment analyses in our previous paper^22^. Between *C. arietinum* and *C. reticulatum*, there is no substantial inversion or alteration in genomic organization. The *C. reticulatum* cp genome showed maximum similarity to the *C. arietinum* cp genome, and this result demonstrated a positive correlation with the megaBLAST result. There are no significant structural differences in gene homology and organization between *C. arietinum* and *C. reticulatum*. These results are in line with previous research that performed the MAUVE analysis in various species or genera like *Cicer*^36,37^.

Codon usage frequency has an important impact on the evolutionary process of the chloroplast organelle, and the GC ratio is one of the factors that influence the preferences^38,39^. Relative synonymous codon usage values (RSCU) describe the ratio between the observed frequency and the expected value of a codon for an amino acid synthesis^40,41^ If the value of RSCU = 1, then there is no bias for the codon used. If the value of RSCU > 1, the usage frequency of the codon is higher than each synonymous codon^42^. The value of RSCU < 1 indicates that the usage frequency of the codon is less than the other codons^43^. Due to the high resemblance between *C. reticulatum* and *C. arietinum* chloroplastic genetic code, the codon usage frequency, and RSCU ratios also have been found quite similar. These findings are also akin to the IR-lacking clade species^44^. We also observed an identical pattern among *C. reticulatum*, *C. echinospermum,* and *C. arietinum* regarding the amino acid compositions encoded in the chloroplast genome. The limited variations in these three cp genomes did not significantly affect the number of amino acid distributions from the chloroplast genome and the overall protein content of these three species.

Inside genomes, there are a large number of simple repeated units (aka SSRs) that are distributed across the genome^45,46^. Analyzing these units can offer a perspective on genome polymorphism level, genetic diversity, as well as genus-specific potential markers^47^. In general, A and T (A/T repeats) nucleotides are abundant in the cp genome of angiosperms instead of G and C nucleotides (G/C repeats)^48^. As a result of cpSSR analysis for *C. reticulatum*, mononucleotide repeats especially the A/T base motif, are found at high levels. This phenomenon is also seen among the IRLC species such as *C. arietinum* ^24^, *Vigna radiata* ^49^*,* *and Vicia faba* ^50^. Moreover, the forward repeat type was the most abundant, and the complement repeat type was the least abundant in *C. reticulatum*’s cp genome. These repeat types and motifs are mostly found in the LSC region instead of the SSC and IR region, like the other species that belong to the IRLC family and the angiosperms^44,51^. To determine the nucleotide diversity among the chloroplast genomes, sliding window analysis was performed with DNAsp v6.11.01 software. Nucleotide variability (Pi ratio) calculations included four species (*C. reticulatum, C. arietinum, C. echinospermum, L. culinaris*) based on their whole chloroplast genome sequence. The rationale behind using the *L. culinaris* cp genome in the divergent hot spot analysis is to identify distinctive regions within the *Cicer* genus, even in comparison to their closest species. Six highly variable regions with a Pi value greater than 0.1 were detected using a sliding window analysis (Fig. 5). The highest Pi value was 0.24583, and it was found in the *clpP* gene. Subsequently, the region of the *ycf1* gene, which is quite close to the *clpP* gene region, showed a Pi value of 0.24278. In the analysis of divergent regions, the *ycf1* gene was mostly found as the main divergent region in the cp genomes^52^. The rest of the peaks belong to the *psbI-psbK* (0.24000), *psaJ-rps12* (0.23806), *accD* (0.21722), and *rps15* (0.14278) coding regions. These regions showed maximum differences in terms of nucleotides. Also, in other studies, some of these six regions have been identified as regions with high variability^53^. Four highly variable sites were found in the LSC region: *clpP, rps12-psaJ, psbI-psbK,* and *accD; ycf1* was found in the IR region, and rps15 was found in the SSC region. These nucleotide differences suggest a high potential to be used as markers to distinguish species at the genus level^54^. Among the angiosperms and land plants, several potential markers are specific to chloroplasts like *ycf1, ycf2, rbcL,* and *matK,* and these informative regions reveal the evolutionary relationships^55,56^.

Phylogenetic trees can be used to examine the species' evolutionary relationships based on a specific genomic region or the whole genome sequence^57,58^. In this study, the whole chloroplast genome sequence of four *Cicer* species and 28 different species was used to observe the phylogenetic position of *Cicer* species. *C. reticulatum, C.bijugum C. echinospermum,* and *C.arietinum* separated from a branch in the tree and formed a separate group (Fig. 6.). This branch node had a high bootstrap value like the other nodes in the tree, and these higher bootstrap values indicate that the tree is well-supported. The positions of *Cicer* species in the phylogenetic tree were found to be consistent as compared to previous studies^44^. As expected, *Cicer* species showed a closer branching to*, Lens culinaris, Lathyrus tuberosus,* and *Oxytropis splendens* species because of being in the same IR-lacking clade, and *Arabidopsis thaliana* formed a separate branch as an outgroup. Our findings support previous findings based on cp-genomes regarding the evolutionary relationships in IRLC^50,59^.

## Conclusion

This study provides the whole chloroplast genome sequence of *Cicer reticulatum*, which is known as a progenitor type of cultivated form, *Cicer arietinum*. After extracting the chloroplast DNA in a high molecular weight (HMW), the cpDNA was sequenced directly in the NGS platform (DNA Nanoball), and this perspective provided a high-accuracy genomic sequence for further analysis. These three *Cicer* species were found remarkably to be similar and preserved regarding the gene order and genomic structure. With the comparative sequence diversity analysis, highly variable sites were identified, resulting in several potential markers for examining the evolutionary relationships among species or species identification. In conclusion, the information obtained from this study constitutes a significant source of information for the genomic research on the *Cicer* genus as it furnishes the benefits of obtaining high-quality cpDNA sequences and gives a standpoint on chloroplast genome engineering research.

## Materials and methods

### Plant material and chloroplast DNA extraction

The seeds of *Cicer reticulatum* were collected from the southeastern part of Turkey called Kesentaş. The seeds collected by Prof. Abdullah Kahraman and the specific genotype of the specimen were registered in herbarium at the Harran University, Faculty of Agriculture, Department of Field Crops, with the validated voucher ID "Kesen_077". The species was identified by Prof. Abdullah Kahraman, one of the co-authors of the paper. The seeds of *C. reticulatum* are stored in the Harran University herbarium for further applications. This specimen is not classified as an endemic or protected plant; consequently, permission was not required to obtain it. The plantation of *C. reticulatum* seeds was made in the experimental plantation field of the Ege University, Faculty of Agriculture in Izmir, Turkey following national and international legislation and regulations. In the study, 25 seeds were planted by leaving ~ 10 cm space between the seeds and ~ 40 cm between the rows. A total of 20 g of fresh, and green leaves of *C. reticulatum* were harvested and stored in a dark environment at 4 °C for 72 h to minimize starch accumulation. A high salt-based chloroplast DNA extraction protocol was followed according to the protocol^60^. The isolated cpDNA was quantified by a spectrophotometer (NanoDrop ND 1000, Thermo Scientific, USA) and visually inspected with agarose gel electrophoresis with 0.8% agarose gel. The isolated cpDNAs were stored at − 80 °C.

### Chloroplast genome sequencing and data processing

50 µl of *C. reticulatum* cpDNA was sent to the Beijing Genome Institute (BGI, Hong Kong, China), and sequenced. Whole Genome Sequencing (WGS) was performed using the DNA Nanoball Sequencing technique. As a summary of the sequencing method, 1 μg genomic DNA was randomly divided into fragments by Covaris and was size-selected by Agencourt AMPure XP-Medium kit, aiming for 200–400 bp fragments. Adapter ligation was completed by adenylation at the 3' end of these fragments. PCR was used to amplify the fragments and with the help of the Agencourt AMPure XP-Medium kit, the PCR products were purified. The final library of single-stranded circular DNAs (sscirDNA) consisted of denatured PCR products with splint oligo sequence. The next step comprises transferring these ssCirDNA molecules into the DNA Nanoball (DNB) which produces approximately 300 copies of cpDNA. With the DNA nanochip technology, these copied cpDNAs were charged into the nanoarrays. Finally, 150 bp paired-end reads were generated with combinatorial Probe-Anchor Synthesis (cPAS). Raw data assembly was performed with Organelle (v1.7.4.1) (https://github.com/Kinggerm/GetOrganelle) software and *C. arietinum* whole cp genome (NC_011163) was used as the reference genome. For the annotation of the chloroplast genome, GeSeq online software was used^61^. Additionally, the circular chloroplast genome map of *C. reticulatum* was demonstrated with the Organellar Genome DRAW online tool^62^. The final whole chloroplast genome sequence of *C. reticulatum* has been deposited to the European Nucleotide Archive (ENA) database under the accession number PRJEB47534.

### Comparative analysis

The mVISTA online tool was used for comparative analysis between *C. reticulatum, C. echinospermum* (ERS7635402), and *C. arietinum* (NC_011163) to reveal differences in genome content and structure among the species^63^. Additionally, the comparative analysis with the MAUVE Alignment was conducted with default parameters to obtain gene homology and gene order^64^.

### Codon usage frequency

Codon usage frequency and relative synonymous codon usage (RSCU) values were calculated with the MEGA-X software^65^. Visualization of these values was performed with R programming using the “ggpubr” package as a plot.

### cpSSRs and repetitive sequence analysis

For the identification of chloroplast simple sequence repeats and repetitive regions, the MIcroSAtellite identification tool (MISA), and REPuter were used. MISA-web tool was utilized to detect the cpSSRs and the minimum criterion was selected as follows, ≥ 10 repeat units for mononucleotide, ≥ 5 repeats units for dinucleotide, ≥ 5 repeats units for trinucleotide, and ≥ 3 repeats unit for tetranucleotide, pentanucleotide, and hexanucleotide, separately^66^. Repetitive sequences were detected using the REPuter online tool with the parameters of min repeat size 30 bp and hamming distance 3 and classified as forward, reverse, palindromic, and complement repeats^67^.

### Divergent hotspot identification

*C. reticulatum, C. arietinum, C. echinospermum,* and *L. culinaris* chloroplast genomes were aligned with MAFFT v7 using default parameters^68^. Taking the alignment file as an input, DnaSP v6.11.01 calculated nucleotide diversity throughout the genomes^69^. To visualize diversity ratios, sliding window analysis was conducted with the parameters for a window length of 600 bp, and a step size of 200 bp.

### Phylogenetic analysis

MEGA-X software was used to reveal the phylogenetic relationships between the four *Cicer* species (*C. reticulatum, C. arietinum* (NC 011,163.1), *C. echinospermum* (ERS7635402)*, C. bijugum*), other Leguminosae family members, and one outgroup species. The complete chloroplast genomes of *Arabidopsis thaliana* (MZ323108.1) -as an outgroup-, *Acacia melanoxylon* (CM032338.1), *Prosopis cineraria* (NC 049,133.1), *Pisum sativum* (CM044353.1), *Medicago truncatula* (NC 003,119.8), *Medicago arabica* (OX326973.1), *Phaseolus vulgaris* (CM055726.1), *Vicia sativa* (CM038904.1), *Acacia acuminata* (CM032337.1), *Acacia pycnantha* (CM046707.1), *Aeschynomene evenia* (CM024273.1), *Arachis hypogaea* (NC 037,358.1), *Lathyrus tuberosus* (CM040791.1), *Lens culinaris* (NC 027,152.1), *Oxytropis splendens* (NC 050,860.1), *Wisteriopsis japonica* (MN311163.1), *Wisteria brachybotrys* (MN311167.1), *Wisteria villosa* (MN311172.1), *Wisteria frutescens* (MN311169.1), *Cajanus cajan* (NC 031,429.1), *Vigna radiata* (NC 013,843.1), *Vigna angularis* (NC 021,091.1), *Vigna unguiculata* (NC 018,051.1), *Glycine max* (CM010429.1), *Glycine max* (CM033154.1), *Glycine max* (NC 007,942.1), *Glycine soja* (NC 022,868.1) and *Glycine max* (CM055187.1) were obtained with the AC number from the NCBI database. We included the chloroplast genome of the widely studied model species *Arabidopsis thaliana*, which is also an angiosperm, in the phylogenetic tree to emphasize relative diversity within legume species. In phylogenetic analysis, we have only focused on coding regions of the known well-annotated genes. For this, we used the GenBank annotation of *Cicer arietinum* from NCBI and sliced the aligned sequences of coding regions from their multiple sequence alignment file. Then, we also removed all putative insertion and deletion sites, only allowing single nucleotide variations across species. This resulted in an input with 50,798 bases of alignment out of which 5593 informative variant sites. MAFFT v7 tool was used to align 32 different chloroplast genome sequences. The command line version of MEGA software (v11.0.13) was used to construct the phylogenetic trees using the Maximum Likelihood method with 1000 bootstrap replicates. General Time Reversible (GTR) model with rates of Gamma distributed and Invariant sites (G + I) was used as this suits the best among 24 substitution models tested using jModelTest v2.1.10^70^.

### Supplementary Information

Supplementary Figure 1.

Supplementary Figure 2.

Supplementary Table 1.

## Data Availability

Data is available on demand. The assembled genome sequences and raw sequencing data are accessible in the European Nucleotide Archive (ENA) database under the research accession PRJEB47534 with the sample identification number ERR6787400.
