# Supplementary material for: The complete chloroplast genome of Cicer reticulatum and comparative analysis against relative Cicer species
Source: Sci Rep. 2023 Oct 19;13:17871. doi: 10.1038/s41598-023-44599-1 (PMC10587350; doi:10.1038/s41598-023-44599-1)
Supplement: Supplementary file 1 — Supplementary Figure 1. [file 41598_2023_44599_MOESM1_ESM.pdf]

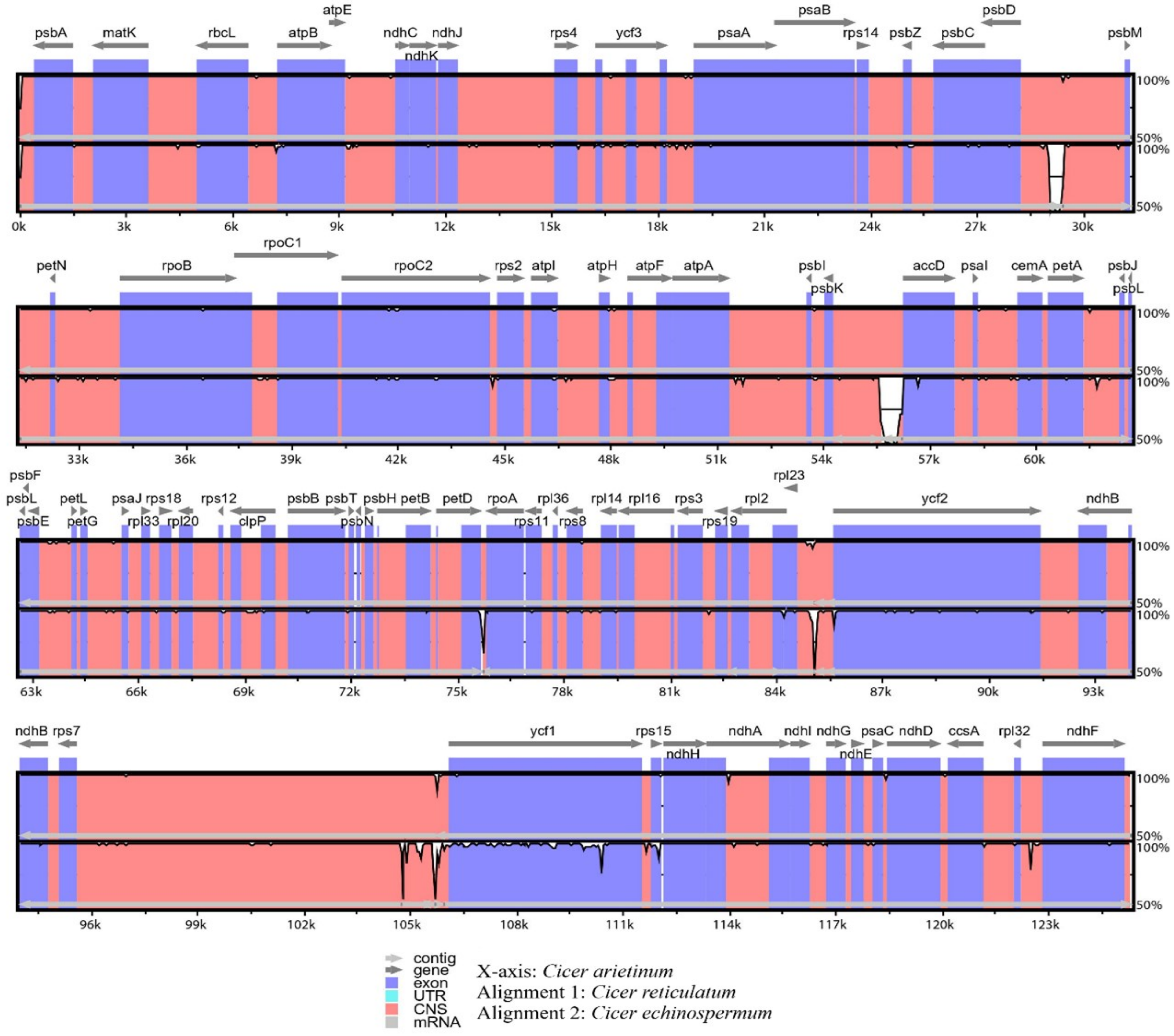

Supplementary figure 1. Comparative analysis of chloroplast genomes of three Cicer species (*Cicer reticulatum, Cicer arietinum, and Cicer echinospermum*). Cicer arietinum is used as a reference genome and is in the baseline. The y-axis displayed the proportion of sequence identity, which ranged from 50% to 100%. Gray arrows indicated the transcriptional orientations of the genes. Non-coding sequences (CNS) were represented by pink bars, whereas exons were represented by purple bars. White peaks are delineated as genomic differences.
