# Supplementary material for: The complete chloroplast genome of Cicer reticulatum and comparative analysis against relative Cicer species
Source: Sci Rep. 2023 Oct 19;13:17871. doi: 10.1038/s41598-023-44599-1 (PMC10587350; doi:10.1038/s41598-023-44599-1)
Supplement: Supplementary file 2 — Supplementary Figure 2. [file 41598_2023_44599_MOESM2_ESM.pdf]

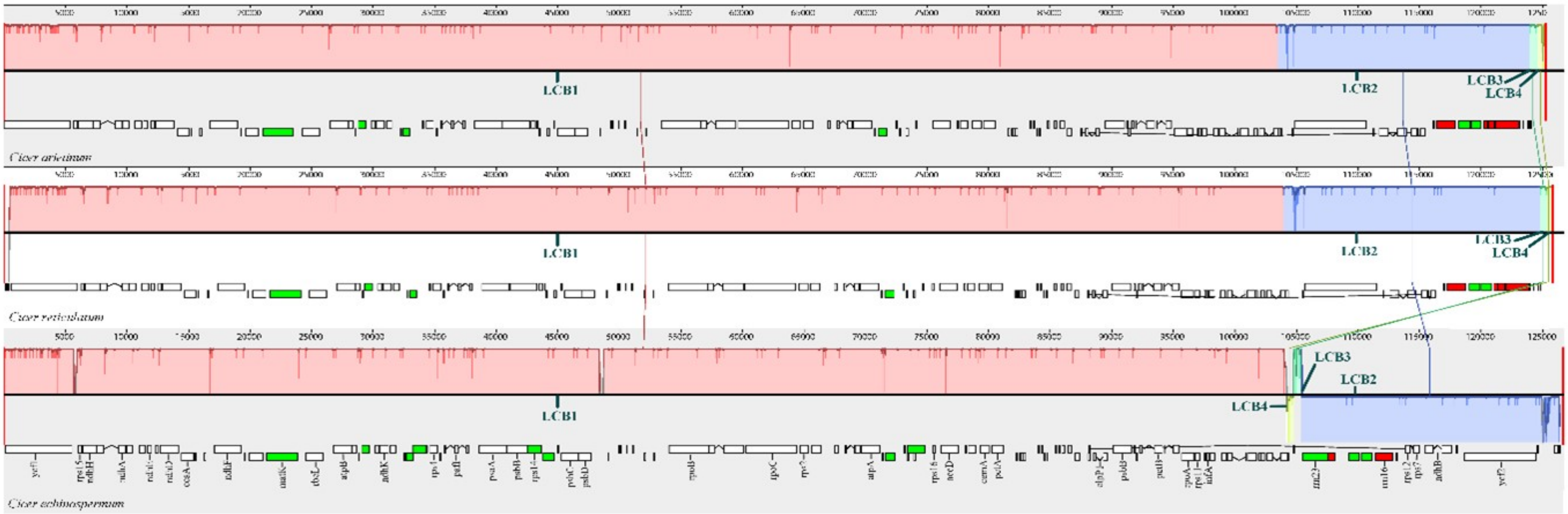

Supplementary figure 2. The gene order and homology between Cicer species are shown in a MAUVE alignment. Histograms that indicate sequence identity with peaks are known as Locally Collinear Blocks (LCBs). Sequences occurring in an inverted orientation are indicated by blocks drawn below the horizontal line. Protein-coding genes, rRNA genes, tRNA genes, and intron-containing tRNA genes are labeled with white, red, black, and green blocks, respectively
