# Supplementary material for: The complete chloroplast genome of Cicer reticulatum and comparative analysis against relative Cicer species
Source: Sci Rep. 2023 Oct 19;13:17871. doi: 10.1038/s41598-023-44599-1 (PMC10587350; doi:10.1038/s41598-023-44599-1)
Supplement: Supplementary file 3 — Supplementary Table 1. [file 41598_2023_44599_MOESM3_ESM.pdf]

Supplementary Table 1. RCSU values of *Cicer reticulatum, Cicer arietinum,* and *Cicer echinospermum* chloroplast genome

Codon	Protein	RSCU Values			Codon	Protein	RSCU Values		
		C. arietinum	*C. reticulatum*	*C. echinospermum*			*C. arietinum*	*C. reticulatum*	*C. echinospermum*
GCU	A	1,21	1,18	1,33	CCA	P	1,32	1,42	1,3
GCA	A	1,41	1,29	1,29	CCU	P	1,15	1,07	1,13
GCC	A	0,74	0,98	0,84	CCC	P	0,85	0,94	0,91
GCG	A	0,64	0,55	0,54	CCG	P	0,68	0,57	0,66
UGU	C	1,24	1,28	1,23	CAA	Q	1,38	1,43	1,38
UGC	C	0,76	0,72	0,77	CAG	Q	0,62	0,57	0,62
GAU	D	1,46	1,38	1,47	AGA	R	2,23	1,94	2,2
GAC	D	0,54	0,62	0,53	AGG	R	1,08	1,04	1,13
GAA	E	1,45	1,47	1,46	CGA	R	1,12	1,09	0,98
GAG	E	0,55	0,53	0,54	CGU	R	0,62	0,67	0,65
UUU	F	1,35	1,34	1,32	CGG	R	0,6	0,76	0,65
UUC	F	0,65	0,66	0,68	CGC	R	0,35	0,5	0,4
GGA	G	1,48	1,48	1,42	UCU	S	1,41	1,35	1,46
GGU	G	1,01	1,07	1,09	UCA	S	1,32	1,45	1,27
GGG	G	0,95	0,89	0,92	UCC	S	1,04	1,04	1,03
GGC	G	0,56	0,57	0,57	AGU	S	0,97	0,86	0,89
CAU	H	1,47	1,41	1,39	UCG	S	0,74	0,72	0,73
CAC	H	0,53	0,59	0,61	AGC	S	0,54	0,59	0,61
AUU	I	1,31	1,26	1,26	UAA	STOP	1,28	1,38	1,35
AUA	I	1,1	1,16	1,13	UGA	STOP	0,94	0,84	0,87
AUC	I	0,59	0,58	0,61	UAG	STOP	0,78	0,78	0,78
AAA	K	1,47	1,44	1,46	ACU	T	1,19	1,17	1,23
AAG	K	0,53	0,56	0,54	ACA	T	1,29	1,19	1,21
UUA	L	1,73	1,66	1,65	ACC	T	0,9	0,98	0,94
UUG	L	1,19	1,09	1,26	ACG	T	0,62	0,65	0,63
CUU	L	1,12	1,15	1,1	GUU	V	1,45	1,44	1,42
CUA	L	0,95	1	0,92	GUA	V	1,27	1,31	1,26
CUC	L	0,56	0,6	0,65	GUC	V	0,66	0,65	0,71
CUG	L	0,46	0,5	0,43	GUG	V	0,62	0,59	0,61
AUG	M	1	1	1	UGG	W	1	1	1
AAU	N	1,45	1,44	1,43	UAU	Y	1,43	1,45	1,42
AAC	N	0,55	0,56	0,57	UAC	Y	0,57	0,55	0,58
